# Supplementary material for: Exploring the awe-some: Mobile eye-tracking insights into awe in a science museum
Source: PLoS One. 2020 Sep 30;15(9):e0239204. doi: 10.1371/journal.pone.0239204 (PMC7526894; doi:10.1371/journal.pone.0239204)
Supplement: S1 Table — (DOCX) [file pone.0239204.s001.docx]

| S1 Table | | | | | | | |
| --- | --- | --- | --- | --- | --- | --- | --- |
| *Correlations (and p-Values) Among Measures, Rotunda* | | | | | | | |
|  | 1 | 2 | 3 | 4 | 5 | 6 | 7 |
| 1. pCenter | – |  |  |  |  |  |  |
|  |  |  |  |  |  |  |  |
|  | | | | | | | |
| 2. pPosters | -0.724 | – |  |  |  |  |  |
|  | (.002) |  |  |  |  |  |  |
|  | | | | | | | |
| 3. pDome | -0.24 | -0.496 | – |  |  |  |  |
|  | (.390) | (.060) |  |  |  |  |  |
|  | | | | | | | |
| 4. Connection | -0.249 | -0.069 | 0.412 | – |  |  |  |
|  | (.372) | (.806) | (.127) |  |  |  |  |
|  | | | | | | | |
| 5. Oppression | 0.152 | -0.209 | 0.104 | -0.108 | – |  |  |
|  | (.587) | (.455) | (.711) | (.701) |  |  |  |
|  | | | | | | | |
| 6. Chills | -0.021 | -0.073 | 0.134 | 0.353 | 0.615 | – |  |
|  | (.942) | (.796) | (.635) | (.196) | (.015) |  |  |
|  | | | | | | | |
| 7. Diminished self | 0.154 | -0.341 | 0.294 | 0.481 | 0.143 | 0.528 | – |
|  | (.583) | (.214) | (.287) | (.069) | (.611) | (.043) |  |
|  | | | | | | | |
| *Note*. pX = proportion of fixations in region of interest X. N = 15. | | | | | | | |
