# Supplementary material for: Exploring the awe-some: Mobile eye-tracking insights into awe in a science museum
Source: PLoS One. 2020 Sep 30;15(9):e0239204. doi: 10.1371/journal.pone.0239204 (PMC7526894; doi:10.1371/journal.pone.0239204)
Supplement: S2 Table — (DOCX) [file pone.0239204.s002.docx]

| S2 Table | | | | | |
| --- | --- | --- | --- | --- | --- |
| *Correlations (and p-Values) Among Measures, U505* | | | | | |
|  | 1 | 2 | 3 | 4 | 5 |
| 1. pUboat | – |  |  |  |  |
|  |  |  |  |  |  |
|  | | | | | |
| 2. Connection | -.313 | – |  |  |  |
|  | (.237) |  |  |  |  |
|  | | | | | |
| 3. Oppression | .323 | -.500 | – |  |  |
|  | (.222) | (.048) |  |  |  |
|  | | | | | |
| 4. Chills | .095 | .317 | .052 | – |  |
|  | (.728) | (.232) | (.848) |  |  |
|  | | | | | |
| 5. Diminished self | .489 | -.049 | .207 | .497 | – |
|  | (.054) | (.857) | (.442) | (.050) |  |
|  | | | | | |
| *Note*. pUboat = proportion of fixations in U505 region of interest. N = 15. | | | | | |
